# Osteoarthritis related epigenetic variations in miRNA expression and DNA methylation

**DOI:** 10.1186/s12920-023-01597-6

**Published:** 2023-07-11

**Authors:** Lingpeng Jin, Jun Ma, Zhen Chen, Fei Wang, Zhikuan Li, Ziqi Shang, Jiangtao Dong

**Affiliations:** 1 Department of Orthopedic Surgery, The Third Hospital of Hebei Medical University, Shijiazhuang, Hebei 050051 China; 2 Hebei Medical University-National University of Ireland Galway Stem Cell Research Center, Hebei Medical University, Shijiazhuang, Hebei 050017 China

**Keywords:** Epigenetics, DNA methylation, MicroRNA, Bioinformatics, Osteoarthritis

## Abstract

Osteoarthritis (OA) is chronic arthritis characterized by articular cartilage degradation. However, a comprehensive regulatory network for OA-related microRNAs and DNA methylation modifications has yet to be established. Thus, we aimed to identify epigenetic changes in microRNAs and DNA methylation and establish the regulatory network between miRNAs and DNA methylation. The mRNA, miRNA, and DNA methylation expression profiles of healthy or osteoarthritis articular cartilage samples were downloaded from Gene Expression Omnibus (GEO) database, including GSE169077, GSE175961, and GSE162484. The differentially expressed genes (DEGs), differentially expressed miRNAs (DEMs), and differentially methylated genes (DMGs) were analyzed by the online tool GEO2R. DAVID and STRING databases were applied for functional enrichment analysis and protein-protein interaction (PPI) network. Potential therapeutic compounds for the treatment of OA were identified by Connectivity map (CMap) analysis. A total of 1424 up-regulated DEGs, 1558 down-regulated DEGs, 5 DEMs with high expression, 6 DEMs with low expression, 1436 hypermethylated genes, and 455 hypomethylated genes were selected. A total of 136 up-regulated and 65 downregulated genes were identified by overlapping DEGs and DEMs predicted target genes which were enriched in apoptosis and circadian rhythm. A total of 39 hypomethylated and 117 hypermethylated genes were obtained by overlapping DEGs and DMGs, which were associated with ECM receptor interactions and cellular metabolic processes, cell connectivity, and transcription. Moreover, The PPI network showed COL5A1, COL6A1, LAMA4, T3GAL6A, and TP53 were the most connective proteins. After overlapping of DEGs, DMGs and DEMs predicted targeted genes, 4 up-regulated genes and 11 down-regulated genes were enriched in the Axon guidance pathway. The top ten genes ranked by PPI network connectivity degree in the up-regulated and downregulated overlapping genes of DEGs and DMGs were further analyzed by the CMap database, and nine chemicals were predicted as potential drugs for the treatment of OA. In conclusion, TP53, COL5A1, COL6A1, LAMA4, and ST3GAL6 may play important roles in OA genesis and development.

**Supplementary Information:**

The online version contains supplementary material available at 10.1186/s12920-023-01597-6.

## Introduction

Osteoarthritis (OA), a prevalent degenerative disease, is characterized by reduced cartilage, synovial inflammation, and osteophyte formation [1–3], which seriously affects human health and quality of life. The pathogenesis of OA is complex and overlapping, including inflammation, mechanical overloading, metabolic imbalances, and cell senescence [4]. Although the understanding of OA development has made great progress, the exact pathogenesis of OA is still largely unknown.

Several studies have recently identified epigenetic modification as a critical regulator involved in OA pathogenesis [5–7]. Epigenetic modifications include DNA methylation, histone modification, and no-coding RNAs, which are closely related to each other and affect patterns of protein syntheses. Interference with epigenetic modifications may lead to dysfunction.

MicroRNAs (miRNAs) are a class of non-coding RNAs containing 21–23 nucleotides that regulate post-transcriptional gene expression in various signaling pathways and biological processes by degrading mRNA or inhibiting translation [8]. Strong evidence supports miRNAs as potential biomarkers and therapeutic targets in osteoarthritis [9, 10]. miR-146a-5p has been discovered to be up-regulated in serum samples from OA patients in several studies and has great potential to serve as an early screening and diagnostic tool for OA due to the less invasive operation of serum sample acquisition [11, 12]. Meanwhile, intra-articular injection of miR-146a-5p antagomir can also inhibit the apoptosis of knee chondrocytes and promote autophagy in OA mice, which provides a potential treatment for OA [13].

DNA methylation is another epigenetic modification that is closely related to the homeostasis of cartilage. For example, increased expression of genes encoding cartilage degradation enzymes was associated with the demethylation of specific CpG sites within these gene promoters in OA cartilage [14]. With the development of DNA methylation sequencing technology, more differential methylation sites (DMS) in OA cartilage have been discovered gradually. Han et al. identified 249 hypermethylated sites and 96 hypomethylation sites in OA cartilage, and 8 genes were identified as potential new biomarkers of OA through functional analysis of differential methylated genes [15]. In addition, Yi et al. identified 2170 DMS between OA and normal cartilage [16].

Up to now, multiple studies have reported aberrant DNA methylation and abnormal expression of miRNAs in OA. However, a comprehensive regulatory network for OA-related microRNAs and DNA methylation modifications has yet to be established. Therefore, we designed this study following the methods of Huang et al. [17], and systematically analyzed mRNA and miRNA expression and DNA methylation microarray data to confirm further the core genes and pathways associated with epigenetic alterations in the regulation of OA.

## Materials and methods

### Microarray data collection

The GEO database was used to obtain the mRNA expression profile microarrays (GSE169077), microRNA profiling (GSE175961), and gene methylation profile data sets (GSE162484). The GSE169077 data set (platform: GPL96 Affymetrix Human Genome U133A Array) included human cartilage samples from 5 normal controls and 6 OA patients. The GSE175961 data set (platform: GPL20712 Agilent-070156 Human miRNA) included cartilage samples of 3 normal controls and 3 OA patients. The GSE162484 data set (platform: GPL13534 Illumina HumanMethylation450 BeadChip) included cartilage samples of 5 normal controls and 5 OA patients.

### Identification of DEGs, DEMs, and DMPs

Through GEO2R online tool, differentially expressed mRNAs (DEGs), miRNAs (DEMs), and methylated CpG probes (DMPs) were identified. DEGs, DEMs, and DMPs were filtered out using the following criteria: *p* < 0.05 and | t |> 2.

### DEMs target genes prediction and miRNA-mRNA regulation network construction

The DEMs were imported to the miRWalk 3.0 online database, which consists of three databases: miRDB, Targetscan, and miRTarBase [18]. miRTarBase is the key database, and all data have been verified experimentally. Genes predicted by miRTarBase and one of the other two databases were considered the target genes of DEMs [19]. Following the alignment of DEMs and DEGs, the Cytoscape software (v 3.9.1) was used to visualize the miRNA-mRNA regulatory network [20].

### Enrichment analysis of function and pathway

To identify the function of the overlapped genes of DEG and other data sets, Gene Ontology (GO) analysis and Kyoto Encyclopedia of Genes and Genomes (KEGG) pathway enrichment analysis [21–23] were conducted by DAVID [24], and the *P* < 0.05 was set as the screening condition.

### Construction and module analysis of protein-protein interaction (PPI) network

The upregulated-hypomethylation genes and downregulated-hypermethylation genes were input into the STRING database to construct a PPI network. Cytoscape was used to visualize the PPI network, and molecular complex detection (MCODE) plug-in was used to screen the modules in the PPI network [25, 26].

### Prediction of potential drugs

The CMap database is widely used in pharmacogenomics research to reveal the functional links between small molecule compounds, genes, and diseases [27, 28]. Compounds with connectivity scores < -90 were selected as potential drugs that might alleviate or inhibit the OA process, and the structures were obtained by the PubChem database [29, 30].

## Results

### Data description, probe screening, and annotation

**Fig. 1:**
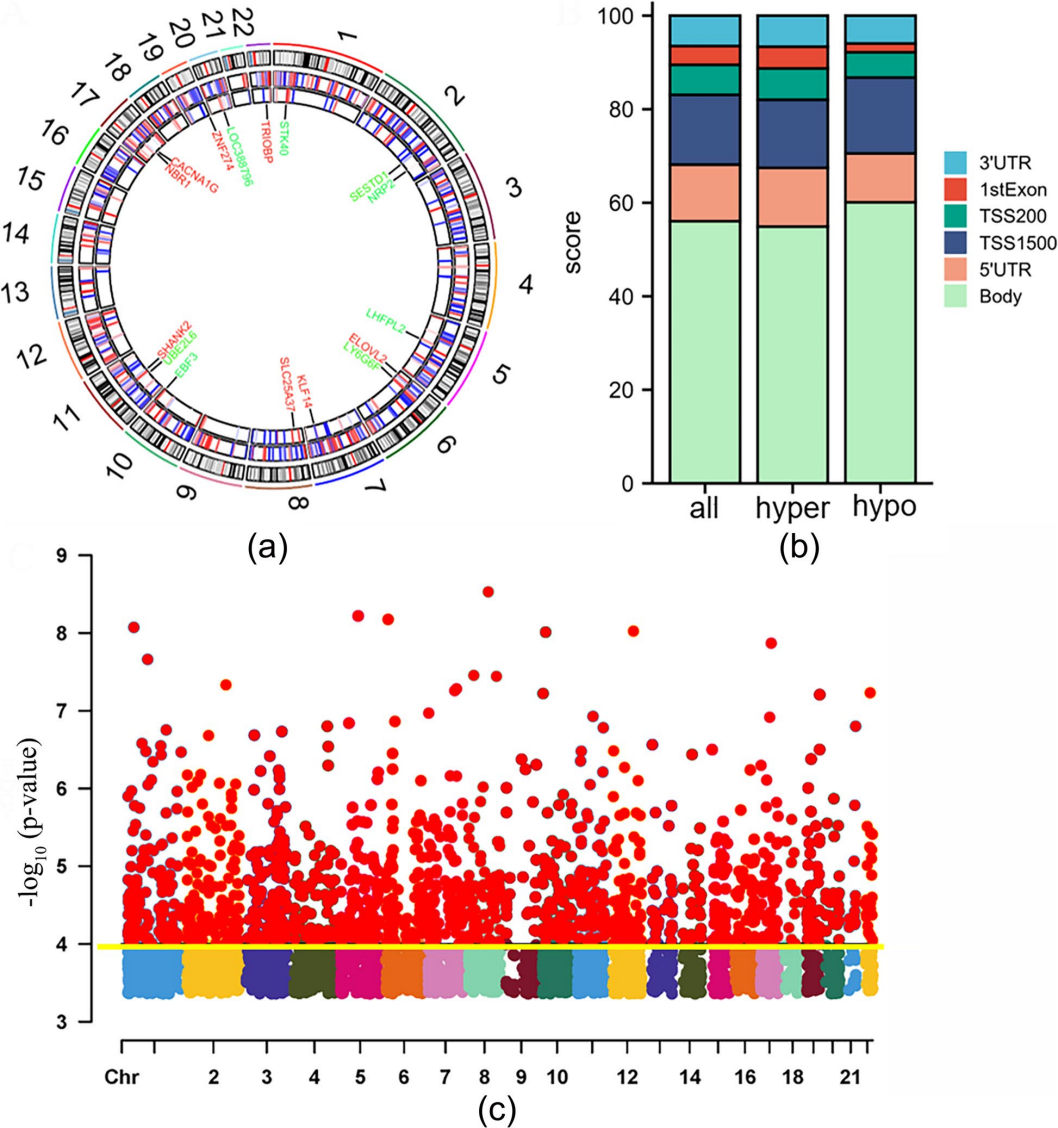
The distribution of differential DNA methylation. **a** Circus plot showing CpGs. The chromosomes are arranged clockwise in the outermost circle, excluding the X and Y chromosomes. Genes marked green and red correspond to the top 8 hypomethylated and hypermethylated genes, respectively. The heat maps of the differentially methylated CGs are shown in the two innermost circles. **b** Bar chart of differentially methylated CpGs in each genomic region. **c** Manhattan plot displaying epigenome-wide association results, and -log_10_ (*p*-value) was labeled with the yellow line

**Fig. 2:**
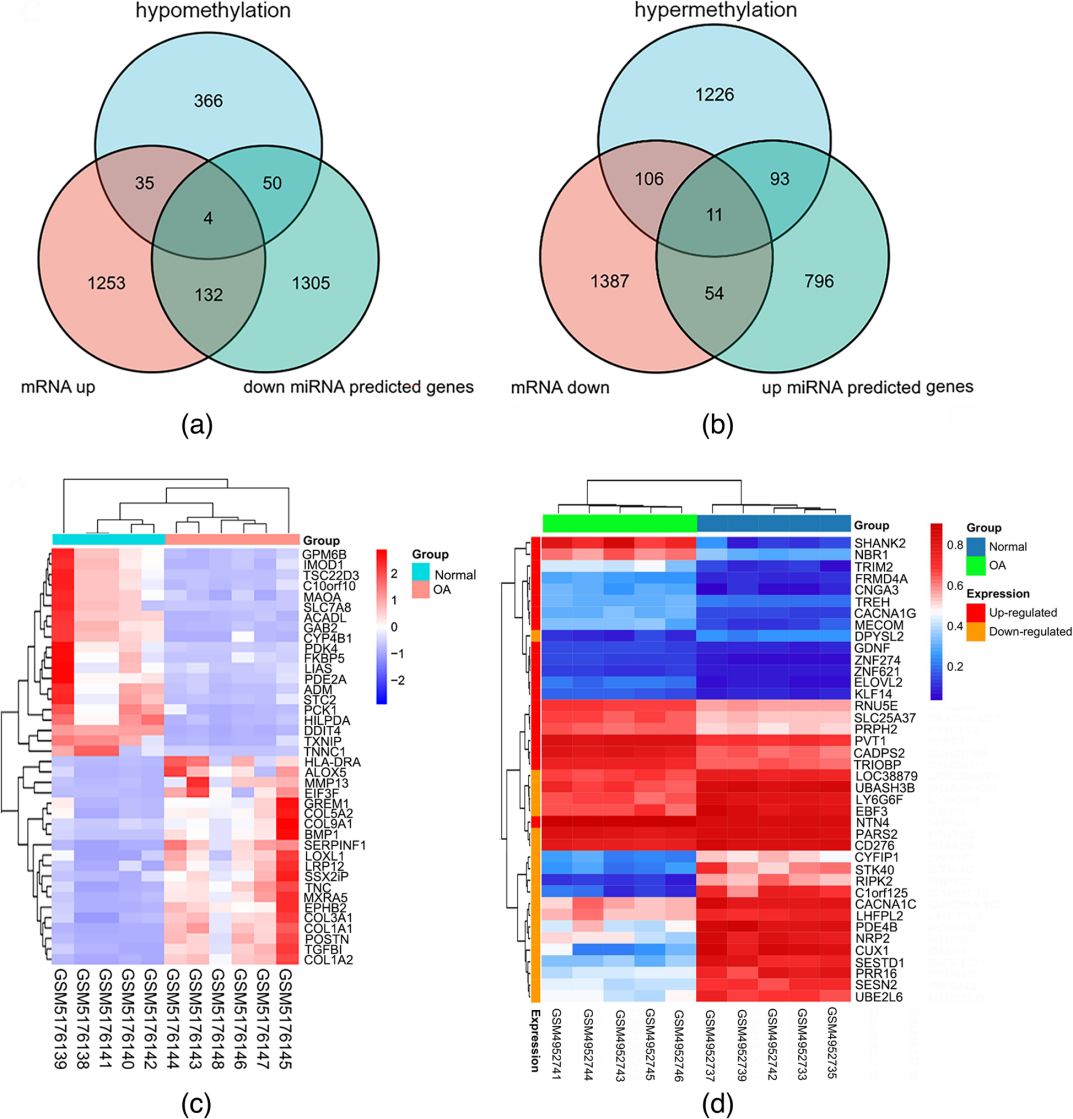
**a** 4 up-regulated genes regulated by both low expression miRNA and hypomethylation were identified by Venn graph. **b** 11 down-regulated genes regulated by both high expression miRNA and hypermethylation were identified by the Venn graph. **c** Heat map of the top 20 up-regulated and top 20 downregulated DEGs of the GSE169077 mRNA expression profile microarray. **d** Heat map of top 20 hypermethylation and 20 hypomethylation genes of GSE162484.

In GSE169077, 2982 differentially expressed mRNAs were screened out, including 1424 up-regulated DEGs and 1558 down-regulated DEGs. In addition, 5 DEMs with high expression and 6 DEMs with low expression were screened out in the miRNAs data set GSE175961. Concerning the GSE162484 gene methylation microarray, 2676 hypermethylated CpG sites located within 1436 genes and 853 hypomethylated CpG sites located within 455 genes have been identified. The distribution of differentially methylated CpG sites located on each autosomal chromosome was demonstrated on the circus plot (Fig. 1a). The proportional distribution of differential methylated CpG sites located in six different genomic subregions is presented in Fig. 1b. In addition, the Manhattan plot showed that differential methylation genes (DMGs) are evenly scattered on autosomes (Fig. 1c). Eventually, 136 up-regulated and 65 downregulated genes were identified by overlapping DEGs and DEMs predicted target genes (Fig. 2a and b). The heatmaps demonstrating the top 40 DEGs and DMGs are shown in Fig. 2c and d.

### Up-regulated genes targeted by low expression miRNAs

**Fig. 3:**
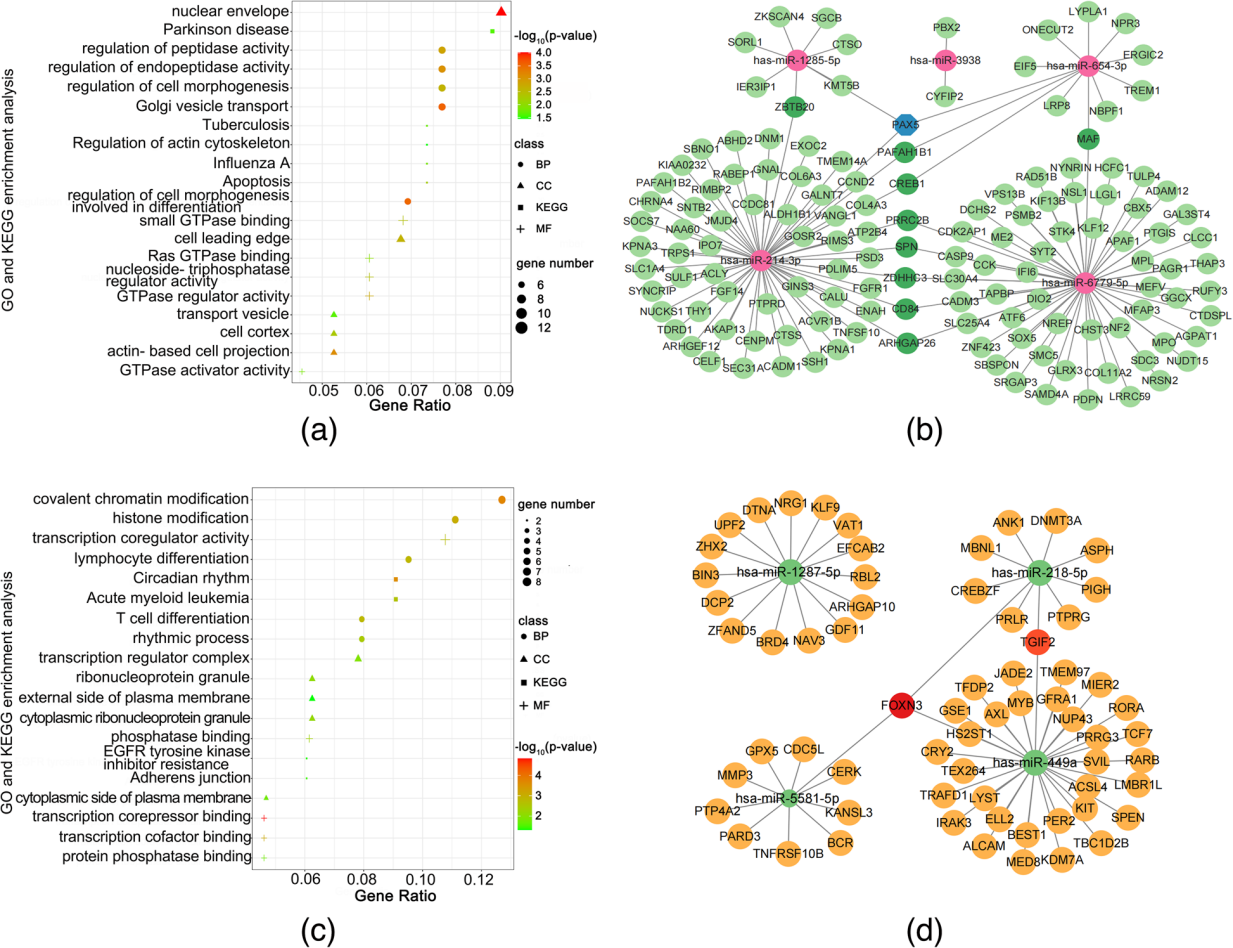
Enrichment analysis and regulatory network of DEGs targeted by miRNAs. **a** The bubble graph showed Go and KEGG pathways enrichment of up-regulated genes targeted by low expression miRNAs. **b** The regulatory network of 5 low expression miRNAs (1 miRNA excluded). **c** The bubble graph showed Go and KEGG pathways enrichment of down-regulated genes targeted by high expression miRNAs. **d** The regulatory graph of 4 high expression miRNAs (1 miRNA excluded)

Figure 2a shows that 136 overlapping up-regulated genes were simultaneously targeted by low expression miRNAs. GO analysis showed that these genes were predominantly enriched in GO terms such as the Golgi vesicle transport, nuclear envelope, and small GTPase binding. Pathways including Apoptosis, Influenza A, Tuberculosis, Parkinson’s disease, and regulation of actin cytoskeleton were identified by KEGG pathways enrichment analysis (Fig. 3a and Supplementary Table S1). To further identify key miRNA-mRNA regulatory panoramas in OA progression, a regulatory network was constructed and revealed that PAX5 was targeted by three miRNAs, and ZBTB20, PAFAH1B1, CREB1, PRRC2B, ZDHHC3, SPN, ARHGAP26, CD84, and MAF were targeted by two miRNAs (Fig. 3b).

### Down-regulated genes targeted by high expression miRNAs

Figure 2b demonstrated that 65 down-regulated genes were targeted by high expression miRNA and these genes were predominantly enriched in GO terms such as the covalent chromatin modification, transcription regulator complex, and transcription coregulator activity (Fig. 3c). Pathways, including Circadian rhythm, Acute myeloid leukemia, Adherens junction, and EGFR tyrosine kinase inhibitor resistance, were identified by KEGG pathways enrichment analysis (Fig. 3c and Supplementary Table S1). To further identify key miRNA-mRNA regulatory panoramas in OA progression, a regulatory network was constructed and revealed that FOXN3 was regulated by 3 miRNAs and TGIF2 was regulated by 2 miRNAs (Fig. 3d).

### Highly expressed and hypomethylation genes

**Fig. 4:**
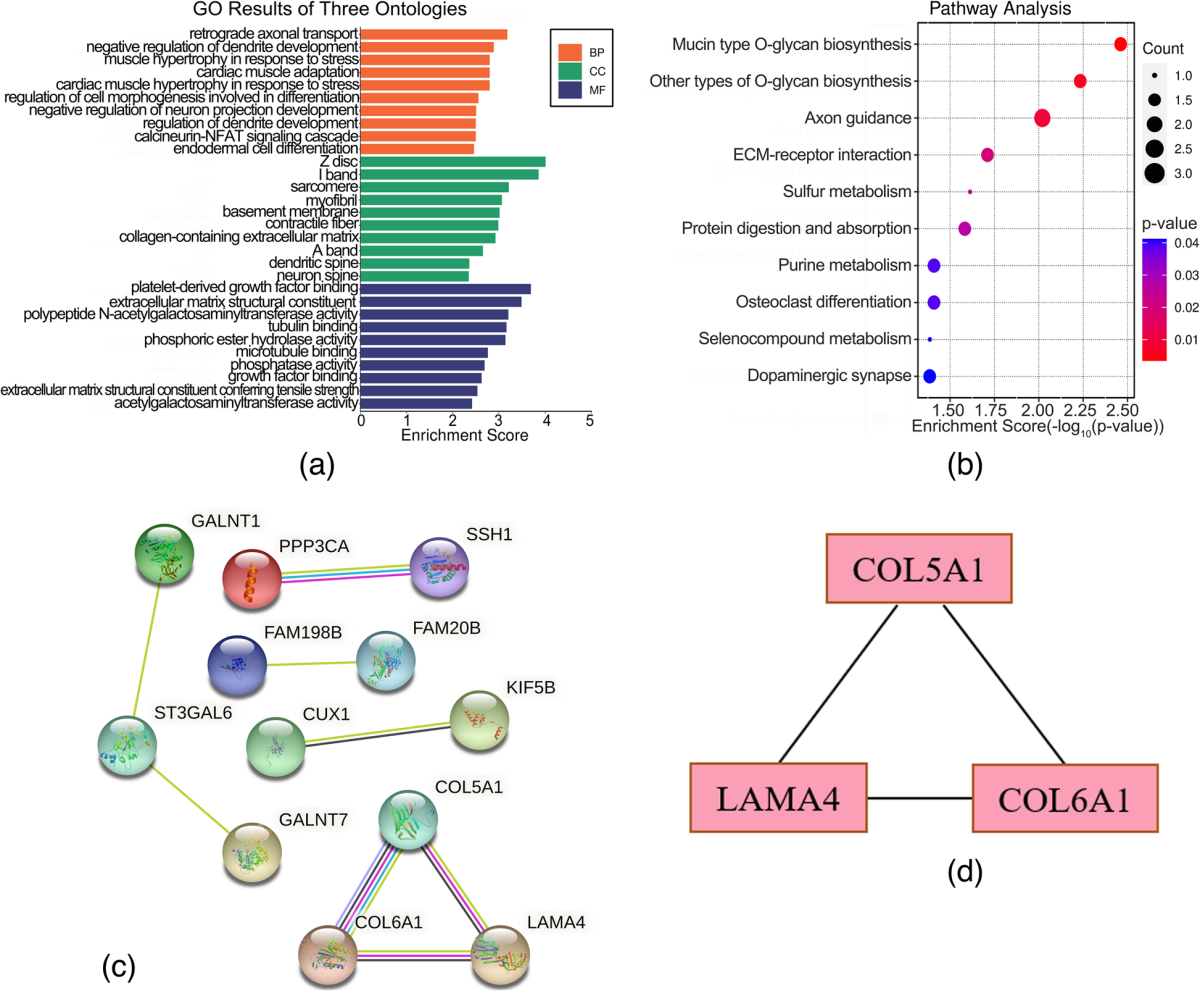
GO and KEGG pathway enrichment analysis and protein-protein interaction network (PPI) of hypomethylation–up-regulated genes. **a** The top 30 enriched GO terms of hypomethylation–up-regulated genes. Enrichment Score=-log_10_(*p*-value). **b** The bubble plot for the top 10 enriched KEGG pathways of hypomethylation–up-regulated genes. **c** PPI network of hypomethylation–up-regulated genes. **d** Top one module of the PPI network for hypomethylation–up-regulated genes

Enrichment analysis showed that enriched genes were related to retrograde axonal transport, Z disc, and platelet-derived growth factor binding (Fig. 4a). KEGG pathway analysis indicated pathways were enriched in Mucin type O-glycan biosynthesis, Other types of O-glycan biosynthesis, Axon guidance, ECM-receptor interaction, Sulfur metabolism, Protein digestion and absorption, Purine metabolism, Osteoclast differentiation, Selenocompound metabolism and Dopaminergic synapse (Fig. 4b and Supplementary Table S2). Totally, 39 nodes and 8 edges were shown in the PPI network (Fig. 4c). The top ten genes ranked by connectivity degree were considered as hub genes, including COL5A1, COL6A1, LAMA4, ST3GAL6, CUX1, FAM198B, FAM20B, GALNT1, GALNT7 and KIF5B (Supplementary Table S3). Among these 10 hub genes, COL5A1, COL6A1, LAMA4 and ST3GAL6 got the highest degree (degree = 2). In addition, the MCODE plug-in was used to identify the important modules found in the PPI network, and the top significant module was selected with 3.00 scores (Fig. 4d).

### Lowly expressed and hypermethylated genes

**Fig. 5:**
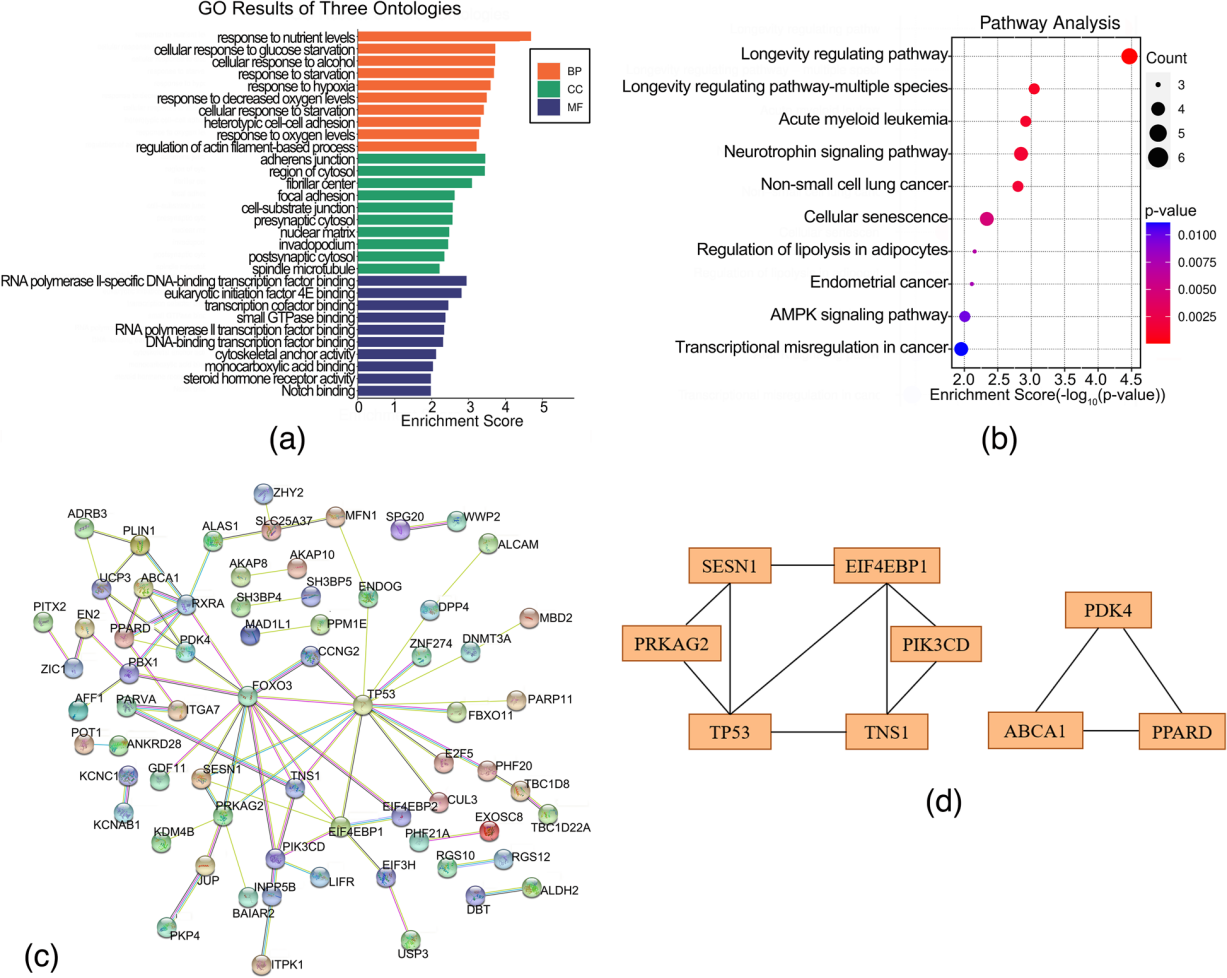
GO and KEGG pathway enrichment analysis and protein-protein interaction network (PPI) of hypermethylated-low-expression genes. **a** The top 30 enriched GO terms of hypermethylated-low-expression genes. Enrichment Score=-log_10_(*p*-value). **b** The bubble plot for the top 10 enriched KEGG pathways of hypermethylated-low-expression genes. **c** PPI network of hypermethylated-low-expression genes. **d** Top two modules of the PPI network for hypermethylated-low-expression genes

The top 30 GO items were identified by enrichment analysis of 117 hypermethylated-low-expression genes, and a barplot was shown in the Fig. 5a. KEGG pathway analysis demonstrated enriched pathways of Longevity regulating pathway, Longevity regulating pathway-multiple species, Acute myeloid leukemia, Neurotrophin signaling pathway, Non-small cell lung cancer Cellular senescence, Regulation of lipolysis in adipocytes, Endometrial cancer, AMPK signaling pathway and Transcriptional misregulation in cancer (Fig. 5b and Supplementary Table S2). Totally, the PPI network showed 115 nodes and 78 edges (Fig. 5c). The top ten ranked genes were considered hub genes based on connectivity degree, including TP53, FOXO3, EIF4EBP1, PRKAG2, RXRA, PIK3CD, PPARD, TNS1, PBX1, and PDK4. TP53 earned the highest degree of the ten hub genes (degree = 15, Supplementary Table S3). Moreover, the top two important modules were selected with 3.6 and 3.0 scores (Fig. 5d).

### DEGs co-regulated by miRNA and DNA methylation

**Fig. 6:**
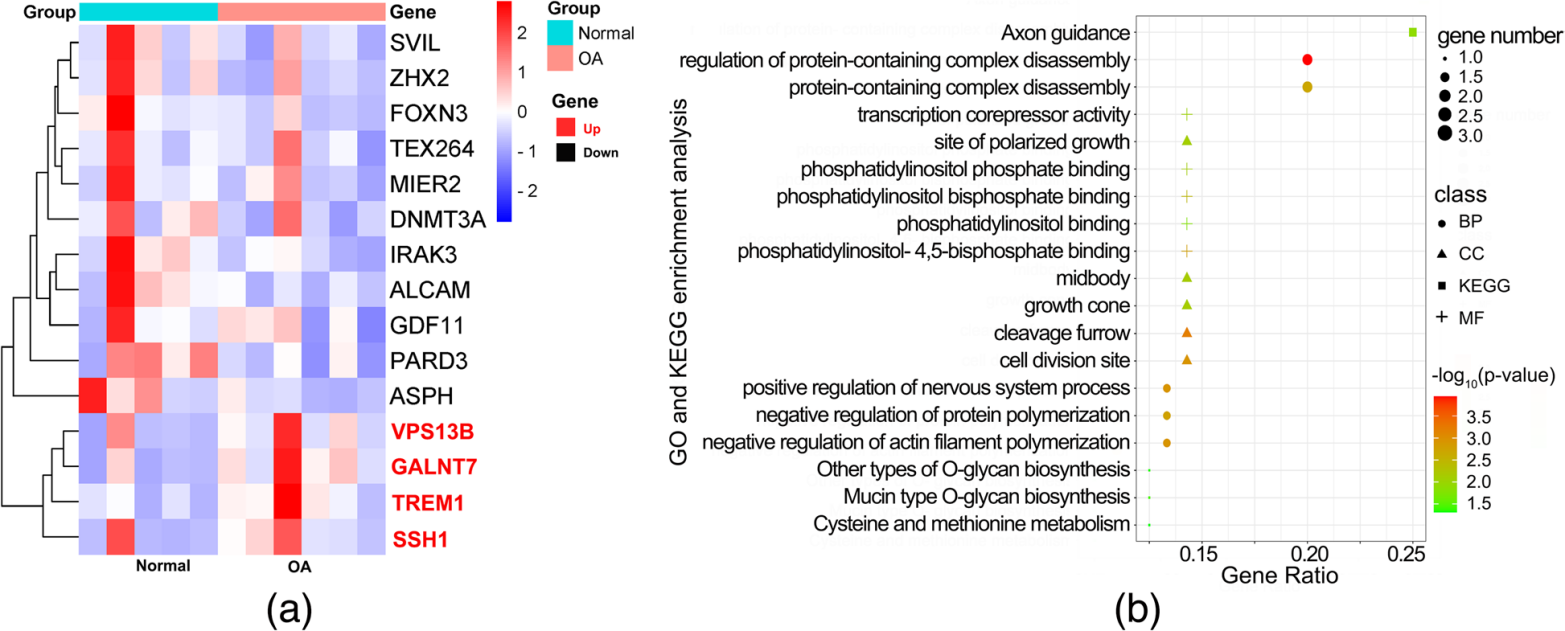
The details of all overlapping genes. **a** Heatmap of the overlapped dual regulated genes. Up-regulated genes are marked in red and down-regulated genes in black. **b** Bubble plot of GO and KEGG enrichment for all overlapping genes

Of note, several DEGs were regulated by both aberrant miRNAs and DNA methylation, which may indicate a more important and complex regulation underlying OA. The expression of SSH1, GALNT7, TREM1, and VPS13B was up-regulated under the modulation of low-expressed miRNAs and hypomethylation. Meanwhile, ALCAM, ZHX2, MIER2, IRAK3, GDF11, PARD3, SVIL, ASPH, FOXN3, DNMT3A, and TEX264 were downregulated by hypermethylation and high expression miRNAs (Fig. 6a).

A summary of DNA methylation sites and their relationships with CpG islands and specific regulation of miRNAs and binding sites was provided in Supplementary Table S4. Simultaneously, Fig. 6b shows the functional and pathway enrichment analysis of these genes.

### Candidate chemicals for OA predicted by CMap analysis

**Fig. 7:**
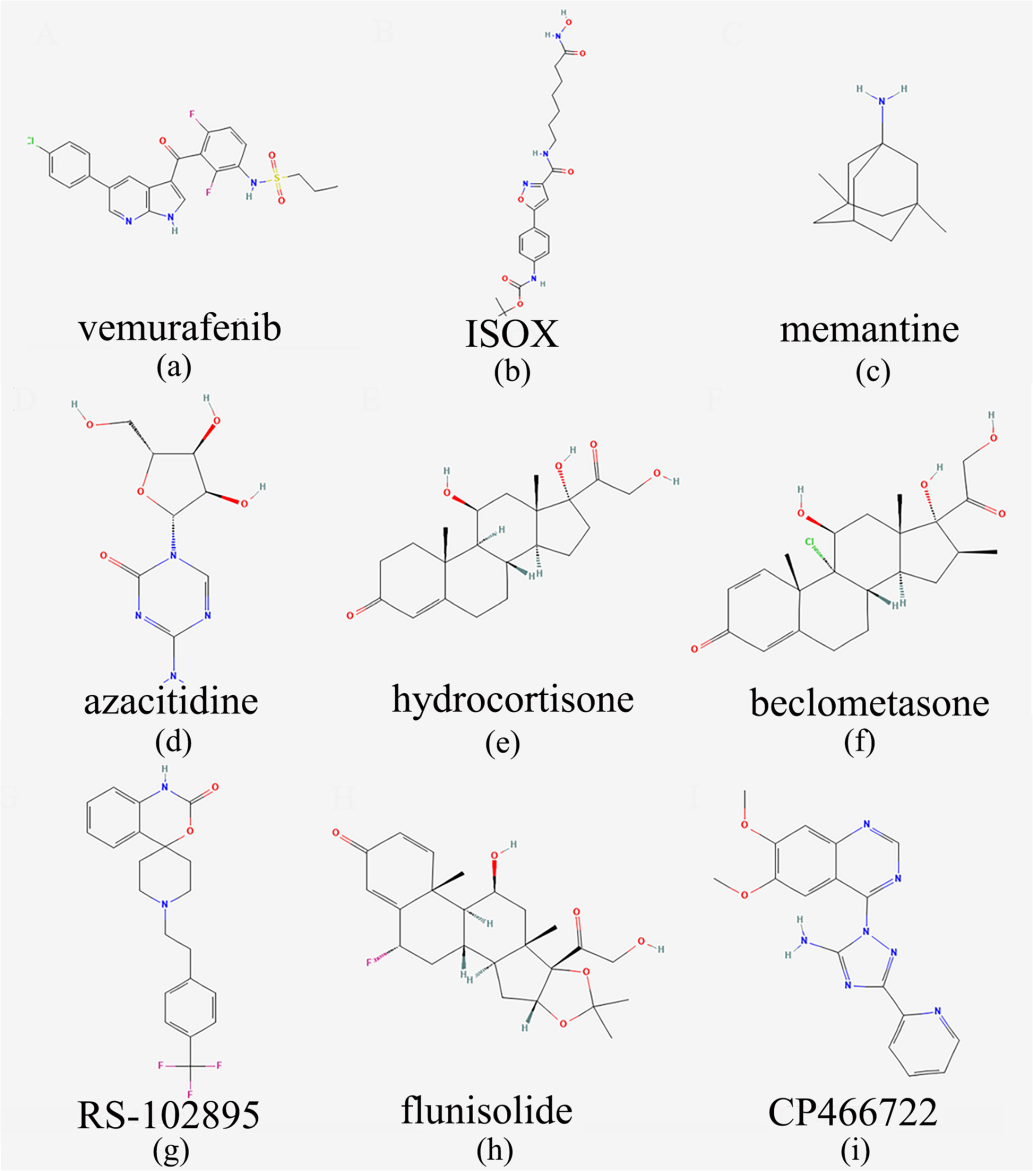
2D structure of the candidate chemicals. **a** vemurafenib; **b** ISOX; **c** memantine; **d** azacytidine; **e** hydrocortisone; **f** beclomethasone; **g** RS-102895; **h** flunisolide; **i** CP466722

The top ten genes ranked by PPI network connectivity degree in up-regulated DEGs and DMGs and downregulated DEGs and DMGs overlapping genes were considered hub genes (Supplementary Table S3) and were sent to the CMap database for further analysis to identify potential chemicals to treat OA. Table 1 lists the top 9 chemicals with the lowest scores that could be potential drugs for OA. The 2D structures of potential compounds obtained from PubChem are shown in Fig. 7.

**Table 1:** Nine candidate chemicals for OA treatment predicted by CMap analysis

Drug name	Score	Description	Target
vemurafenib	-96.86	RAF inhibitor	BRAF, CYP2C19, CYP3A4, CYP3A5, RAF1
ISOX	-96.65	HDAC inhibitor	HDAC6
memantine	-95.35	Glutamate receptor antagonist	GRIN1, CHRFAM7A, CYP2E1, DRD2, GRIN2A, GRIN2B, GRIN3A, HTR3A
azacitidine	-95.24	DNA methyltransferase inhibitor	DNMT1
hydrocortisone	-95.17	Glucocorticoid receptor agonist	ANXA1, NOS2, NR3C1, NR3C2
beclometasone	-94.67	Glucocorticoid receptor agonist	NR3C1, CYP3A5, GPR97, SERPINA6
RS-102895	-94.01	CCR antagonist	CCR2
flunisolide	-93.92	Cytochrome P450 inhibitor	NR3C1
CP466722	-93.91	ATM kinase inhibitor	ATM

## Discussion

Alterations in miRNA expression, as well as DNA methylation, have a significant effect on the initiation and development of OA diseases by altering gene transcriptional activity [31, 32]. Consequently, miRNA expression and DNA methylation pattern alterations can be regarded as useful biomarkers to assist in the diagnosis of OA and potential therapeutic targets for OA therapy [31]. In this study, we systematically analyzed mRNA microarray (GSE169077), miRNAs microarray (GSE175961), and DNA methylation microarray (GSE162484) and compared the expression differences between OA and control cartilage samples. Pivotal genes and pathways that regulate epigenetic alterations in miRNAs and DNA methylation were identified.

miRNAs have been shown to have key roles in chondrocyte development and cartilage homeostasis via negatively regulating gene expression [33]. In the present study, a total of 136 up-regulated and 65 downregulated genes were identified by overlapping DEGs and DEMs target genes. These genes were found to be predominantly enriched in the nuclear envelope and chromatin covalent modification by GO analysis, indicating that nuclear envelope changes and chromatin modifications may play a vital role in OA pathogenesis [34]. Pathways enrichment, including Apoptosis, Influenza A, Tuberculosis, Parkinson’s disease, regulation of actin cytoskeleton, circadian rhythm, adherens junction, and EGFR tyrosine kinase inhibitor resistance were identified by KEGG pathway enrichment analysis. Apoptosis is a highly regulated cell death process characterized by characteristic morphological changes in cellular structures and enzyme - and energy-dependent biochemical processes involved in cell development, homeostasis, and senescence [35–37]. Studies have shown that abnormal chondrocyte apoptosis has a significant effect on the pathogenesis of OA [38, 39]. Further, inhibition of the chondrocyte apoptotic signaling pathway through epigenetic alterations has been shown to reduce the progression of osteoarthritis [40, 41]. The actin cytoskeleton is an important structure by which cells change shape and surface mechanical properties and interact with other cells and the environment. It is involved in several pathophysiological processes, including cancer metastasis, embryonic development, inflammation, and wound repair [42, 43]. In addition, recent studies have shown that the actin cytoskeleton modulates COL1 expression, regulates the production of COL2 and aggrecan fragments, and mediates a fibrogenic/catabolic expression profile [44]. This implies that the actin cytoskeleton can regulate the chondrocyte phenotype and maybe play important roles in the pathological process of OA. Changes in the regulatory signaling pathways of the actin cytoskeleton were also demonstrated in the mouse model of traumatic OA [45]. Circadian rhythm has been identified as the most dysregulated pathway in human OA in articular cartilage [46] EGFR signaling is essential for maintaining superficial chondrocytes during articular cartilage development and homeostasis. Targeting activation of EGFR signaling could be an effective OA therapy [47]. In addition, miRNA-mRNA regulatory network demonstrated that PAX5 and FOXN3 were targeted by three miRNAs, and ZBTB20, PAFAH1B1, CREB1, PRRC2B, ZDHHC3, SPN, ARHGAP26, CD84, MAF, and TGIF2 were targeted by two miRNAs. Among these genes, PAX5 [48], ZBTB20 [49], SPN [50], and CREB1 [51] have been found to regulate the progression of OA, but the role of other genes in OA remains unclear. In addition, the highly expressed hsa-miR-449a, hsa-miR-1287-5p, and lowly expressed has-miR-214-3p and has-miR-6779-5p in OA cartilage tissues targeted more than 15 genes. Among those miRNAs, miR-449a has been reported could promote chondrocytes’ extracellular matrix degradation [52]. Decreased miR-214-3p promoted ECM metabolism and cell apoptosis via activating the NF-κB signaling pathway [53]. A single miRNA is responsible for the regulation of multiple messenger RNAs. Therefore, targeted abnormal expressed miRNA may modulate multiple compensatory pathways, which is an ideal therapeutic strategy for OA [54].

The gene-specific methylation has proved a clear link between methylation and the development of OA. In the present study, we combined and analyzed the DEGs and DMGs and found that these genes were predominantly enriched in retrograde axonal transport, Z disc and platelet-derived growth factor binding, ECM-receptor interaction, cell metabolic processes, cell junctions, and transcription. Similarly, a study on transcriptome data analysis of human knee cartilage (GSE114007) also reported enrichment of the “ECM receptor interaction” KEGG pathway [55]. After establishing the PPI network, - COL5A1, COL6A1, LAMA4, ST3GAL6, and TP53 were the genes with the most connectivity. Analysis of GSE82107 and GSE55235 datasets by Zhu et al. identified COL5A1 as one of the top ten genes [56]. Additionally, Fang et al. also found that COL5A1 and COL6A1 were the top ten hub genes in the analysis of gene expression profile datasets (GSE51588, GSE114007) and gene methylation dataset (GSE64393) [57]. Moreover, COL6A1 deficiency accelerated OA in mice [58]. LAMA4 has been reported to increase in OA cartilage and promote MMP3 transcription [59]. The sialyltransferase ST3GAL6 is highly-expressed in OA chondrocytes [60]. Altered sialylation catalyzed by specific sialyltransferase is involved in the development of OA in several types of research [61, 62]. The role of TP53 gene in OA treatment has been verified in several studies [63–65]. The increased p53 level is associated with clinical OA grades [66]. Inhibition of p53 alleviated OA progression via reducing senescence and apoptosis in chondrocytes [67]. Therefore, these five genes may be potential biomarkers or therapeutic target genes in OA.

Since there are no effective drugs for OA treatment, CMap was used to predict some potential compounds [28, 68]. Nine candidate chemicals were identified from the CMap database. Memantine, a traditional treatment for Alzheimer’s disease, has recently been found to delay OA progression by inhibiting the degradation of extracellular mesenchyme induced by advanced glycation end products [69]. Numerous studies have revealed that histone deacetylases (HDACs) are involved in OA progression, and HDAC inhibitors can inhibit Oprogression [70–73]. Therefore, ISOX, as an HDAC inhibitor targeting HDAC6, may play a potential role in the treatment of OA. In addition, azacitidine, as a DNA methyltransferase inhibitor, may also be a potential drug for the treatment of OA [74, 75]. However, the clinical effects of those candidate chemicals on OA need further investigation.

Of course, our research inevitably has some limitations. First of all, the DEGs, DEM, and methylation data sets included in this study were derived from different cohorts so there was a certain tissue heterogeneity. In addition, the number of biological samples for DEG, DEM and methylation was relatively small.

## Conclusion

In conclusion, this research demonstrated a range of differentially expressed genes in OA closely related to epigenetic modifications of DNA methylation and miRNA expression. Nine compounds were thought to have the potential for OA treatment. Moreover, TP53, COL5A1, COL6A1, LAMA4, and ST3GAL6 may play important roles in OA genesis and development and have the potential to serve as biomarkers for accurate diagnosis and treatment of OA in the future.

## Supplementary Information

**Additional file 1: Supplementary Table S1.** Gene ontology and KEGG pathway analysis of DEGs targeted by altered miRNAs between OA and normal samples.

**Additional file  2: Supplementary Table S2.** Gene ontology and KEGG pathway analysis of DEGs associated with aberrant DNA methylation between OA and normal samples.

**Additional file 3: Supplementary Table S3.** Hub genes with the top 10 degrees of both high expression with hypomethylation and low expression genes with hypermethylation.

**Additional file 4: Supplementary Table S4.** DEGs associated with both specific miRNA and DNA methylation CpG sites between OA and healthy samples.

**Additional file 5: Supplementary Table S5.** The gene list of dual-regulated genes. Left: up-regulated genes with hypomethylation and low miRNA. Right: down-regulated genes with hypermethylation and high miRNA.

## Data Availability

The data (expression profiles: GSE169077, GSE175961, and GSE162484) used in this study are public and available from the GEO database (https://www.ncbi.nlm.nih.gov).
