# Supplementary material for: Osteoarthritis related epigenetic variations in miRNA expression and DNA methylation
Source: BMC Med Genomics. 2023 Jul 11;16:163. doi: 10.1186/s12920-023-01597-6 (PMC10337191; doi:10.1186/s12920-023-01597-6)
Supplement: Supplementary file 1 — Additional file 1: Supplementary Table S1. Gene ontology and KEGG pathway analysis of DEGs targeted by altered miRNAs between OA and normal samples. [file 12920_2023_1597_MOESM1_ESM.docx]

Supplementary Table S1. Gene ontology and KEGG pathway analysis of DEGs targeted by altered miRNAs between OA and normal samples.

| **Category** | **Term** | **GeneRatio** | **Count** | **P value** |
| --- | --- | --- | --- | --- |
| ***Low miRNA targeting up-regulated genes*** | |  |  |  |
| Biological Process | Golgi vesicle transport | 10/130 | 8 | 0.000190637 |
| Biological Process | regulation of endopeptidase activity | 10/130 | 7 | 0.000922014 |
| Biological Process | regulation of peptidase activity | 10/130 | 6 | 0.001408735 |
| Biological Process | regulation of cell morphogenesis | 10/130 | 5 | 0.001555231 |
| Biological Process | regulation of cell morphogenesis involved in differentiation | 9/130 | 5 | 0.003493538 |
| Cellular Component | nuclear envelope | 12/133 | 5 | 0.011272279 |
| Cellular Component | cell leading edge | 9/133 | 4 | 0.007134636 |
| Cellular Component | actin-based cell projection | 7/133 | 4 | 0.008247765 |
| Cellular Component | cell cortex | 7/133 | 4 | 0.047605059 |
| Cellular Component | transport vesicle | 7/133 | 3 | 0.016601888 |
| Molecular Function | small GTPase binding | 9/132 | 7 | 0.001857865 |
| Molecular Function | GTPase regulator activity | 8/132 | 4 | 0.004959502 |
| Molecular Function | nucleoside-triphosphatase regulator activity | 8/132 | 3 | 1.50121E-05 |
| Molecular Function | Ras GTPase binding | 8/132 | 3 | 0.000782174 |
| Molecular Function | GTPase activator activity | 6/132 | 3 | 0.015875272 |
| KEGG Pathway | Parkinson disease | 6/68 | 6 | 0.023519683 |
| KEGG Pathway | Apoptosis | 5/68 | 5 | 0.005499711 |
| KEGG Pathway | Influenza A | 5/68 | 5 | 0.01403254 |
| KEGG Pathway | Tuberculosis | 5/68 | 5 | 0.017175289 |
| KEGG Pathway | Regulation of actin cytoskeleton | 5/68 | 5 | 0.035506897 |
| ***High miRNA targeting down-regulated genes*** | |  |  |  |
| Biological Process | covalent chromatin modification | 8/63 | 8 | 0.000191 |
| Biological Process | histone modification | 7/63 | 7 | 0.000922 |
| Biological Process | lymphocyte differentiation | 6/63 | 6 | 0.001409 |
| Biological Process | T cell differentiation | 5/63 | 5 | 0.001555 |
| Biological Process | rhythmic process | 5/63 | 5 | 0.003494 |
| Cellular Component | transcription regulator complex | 5/64 | 5 | 0.011272 |
| Cellular Component | cytoplasmic ribonucleoprotein granule | 4/64 | 4 | 0.007135 |
| Cellular Component | ribonucleoprotein granule | 4/64 | 4 | 0.008248 |
| Cellular Component | external side of plasma membrane | 4/64 | 4 | 0.047605 |
| Cellular Component | cytoplasmic side of plasma membrane | 3/64 | 3 | 0.016602 |
| Molecular Function | transcription coregulator activity | 7/65 | 7 | 0.001858 |
| Molecular Function | phosphatase binding | 4/65 | 4 | 0.00496 |
| Molecular Function | transcription corepressor binding | 3/65 | 3 | 1.5E-05 |
| Molecular Function | transcription cofactor binding | 3/65 | 3 | 0.000782 |
| Molecular Function | protein phosphatase binding | 3/65 | 3 | 0.015875 |
| KEGG Pathway | Circadian rhythm | 3/33 | 3 | 0.000255 |
| KEGG Pathway | Acute myeloid leukemia | 3/33 | 3 | 0.002459 |
| KEGG Pathway | Adherens junction | 2/33 | 2 | 0.033469 |
| KEGG Pathway | EGFR tyrosine kinase inhibitor resistance | 2/33 | 2 | 0.040672 |
