# Supplementary material for: Osteoarthritis related epigenetic variations in miRNA expression and DNA methylation
Source: BMC Med Genomics. 2023 Jul 11;16:163. doi: 10.1186/s12920-023-01597-6 (PMC10337191; doi:10.1186/s12920-023-01597-6)
Supplement: Supplementary file 2 — Additional file 2: Supplementary Table S2. Gene ontology and KEGG pathway analysis of DEGs associated with aberrant DNA methylation between OA and normal samples. [file 12920_2023_1597_MOESM2_ESM.docx]

Supplementary Table S2. Gene ontology and KEGG pathway analysis of DEGs associated with aberrant DNA methylation between OA and normal samples.

| **Category** | **Term** | **Count** | ***P* value** | ***-log_10_(P* value*)*** |
| --- | --- | --- | --- | --- |
| ***Hypomethylation and up-regulated genes*** | | | |  |
| Biological Process | retrograde axonal transport | 2 | 0.00065826 | 3.181602408 |
| Biological Process | negative regulation of dendrite development | 2 | 0.001297085 | 2.887031447 |
| Biological Process | muscle hypertrophy in response to stress | 2 | 0.001589893 | 2.798632042 |
| Biological Process | cardiac muscle adaptation | 2 | 0.001589893 | 2.798632042 |
| Biological Process | cardiac muscle hypertrophy in response to stress | 2 | 0.001589893 | 2.798632042 |
| Biological Process | regulation of cell morphogenesis involved in differentiation | 4 | 0.002782959 | 2.555493213 |
| Biological Process | negative regulation of neuron projection development | 3 | 0.003125987 | 2.505012841 |
| Biological Process | regulation of dendrite development | 3 | 0.003183532 | 2.497090781 |
| Biological Process | calcineurin-NFAT signaling cascade | 2 | 0.003184525 | 2.496955317 |
| Biological Process | endodermal cell differentiation | 2 | 0.003475798 | 2.458945443 |
| Cellular Component | Z disc | 4 | 9.77676E-05 | 4.00980519 |
| Cellular Component | I band | 4 | 0.000138235 | 3.85938346 |
| Cellular Component | sarcomere | 4 | 0.000612198 | 3.213108238 |
| Cellular Component | myofibril | 4 | 0.000864056 | 3.063458168 |
| Cellular Component | basement membrane | 3 | 0.000967549 | 3.014327022 |
| Cellular Component | contractile fiber | 4 | 0.001030037 | 2.987147127 |
| Cellular Component | collagen-containing extracellular matrix | 5 | 0.001187319 | 2.925432625 |
| Cellular Component | A band | 2 | 0.002224312 | 2.65280439 |
| Cellular Component | dendritic spine | 3 | 0.004375404 | 2.358981813 |
| Cellular Component | neuron spine | 3 | 0.004516325 | 2.345214861 |
| Molecular Function | platelet-derived growth factor binding | 2 | 0.000203498 | 3.691438807 |
| Molecular Function | extracellular matrix structural constituent | 4 | 0.000324823 | 3.488352813 |
| Molecular Function | polypeptide N-acetylgalactosaminyltransferase activity | 2 | 0.000626479 | 3.203093574 |
| Molecular Function | tubulin binding | 5 | 0.000687386 | 3.162799079 |
| Molecular Function | phosphoric ester hydrolase activity | 5 | 0.000722018 | 3.141452089 |
| Molecular Function | microtubule binding | 4 | 0.001740674 | 2.759282676 |
| Molecular Function | phosphatase activity | 4 | 0.002045682 | 2.689161811 |
| Molecular Function | growth factor binding | 3 | 0.002376741 | 2.624018166 |
| Molecular Function | extracellular matrix structural constituent conferring tensile strength | 2 | 0.00292383 | 2.53404795 |
| Molecular Function | acetylgalactosaminyltransferase activity | 2 | 0.00382612 | 2.417241459 |
| KEGG Pathway | Mucin type O-glycan biosynthesis | 2 | 0.003457625 | 2.46122213 |
| KEGG Pathway | Other types of O-glycan biosynthesis | 2 | 0.00583738 | 2.23378203 |
| KEGG Pathway | Axon guidance | 3 | 0.009551143 | 2.01994465 |
| KEGG Pathway | ECM-receptor interaction | 2 | 0.019461371 | 1.71082656 |
| KEGG Pathway | Sulfur metabolism | 1 | 0.024387548 | 1.61283186 |
| KEGG Pathway | Protein digestion and absorption | 2 | 0.02612489 | 1.58294553 |
| KEGG Pathway | Purine metabolism | 2 | 0.038970908 | 1.40925947 |
| KEGG Pathway | Osteoclast differentiation | 2 | 0.038970908 | 1.40925947 |
| KEGG Pathway | Selenocompound metabolism | 1 | 0.041121491 | 1.38593114 |
| KEGG Pathway | Dopaminergic synapse | 2 | 0.041212942 | 1.38496638 |
| ***Hypermethylation and down-regulated genes*** | | | |  |
| Biological Process | response to nutrient levels | 12 | 2.04225E-05 | 4.68989106 |
| Biological Process | cellular response to glucose starvation | 4 | 0.000189388 | 3.72264772 |
| Biological Process | cellular response to alcohol | 5 | 0.000191984 | 3.71673466 |
| Biological Process | response to starvation | 7 | 0.000205678 | 3.68681152 |
| Biological Process | response to hypoxia | 9 | 0.000253981 | 3.59519862 |
| Biological Process | response to decreased oxygen levels | 9 | 0.000323333 | 3.49034963 |
| Biological Process | cellular response to starvation | 6 | 0.000387428 | 3.4118085 |
| Biological Process | heterotypic cell-cell adhesion | 4 | 0.000470478 | 3.32746114 |
| Biological Process | response to oxygen levels | 9 | 0.000518772 | 3.28502342 |
| Biological Process | regulation of actin filament-based process | 9 | 0.000609391 | 3.21510371 |
| Cellular Component | adherens junction | 6 | 0.000353109 | 3.4520907 |
| Cellular Component | region of cytosol | 3 | 0.000363753 | 3.43919347 |
| Cellular Component | fibrillar center | 5 | 0.000812742 | 3.09004746 |
| Cellular Component | focal adhesion | 8 | 0.002393375 | 2.6209892 |
| Cellular Component | cell-substrate junction | 8 | 0.002691078 | 2.57007374 |
| Cellular Component | presynaptic cytosol | 2 | 0.002729057 | 2.56398741 |
| Cellular Component | nuclear matrix | 4 | 0.003349909 | 2.47496701 |
| Cellular Component | invadopodium | 2 | 0.003572448 | 2.44703411 |
| Cellular Component | postsynaptic cytosol | 2 | 0.004521601 | 2.34470773 |
| Cellular Component | spindle microtubule | 3 | 0.006124728 | 2.21291317 |
| Molecular Function | RNA polymerase II-specific DNA-binding transcription factor binding | 7 | 0.001135501 | 2.94481244 |
| Molecular Function | eukaryotic initiation factor 4E binding | 2 | 0.00155257 | 2.80894893 |
| Molecular Function | transcription cofactor binding | 3 | 0.003540181 | 2.45097459 |
| Molecular Function | small GTPase binding | 8 | 0.004237972 | 2.37284189 |
| Molecular Function | RNA polymerase II transcription factor binding | 3 | 0.004611463 | 2.33616125 |
| Molecular Function | DNA-binding transcription factor binding | 7 | 0.004909609 | 2.30895307 |
| Molecular Function | cytoskeletal anchor activity | 2 | 0.007604801 | 2.11891217 |
| Molecular Function | monocarboxylic acid binding | 3 | 0.009259887 | 2.03339433 |
| Molecular Function | steroid hormone receptor activity | 2 | 0.010534084 | 1.97740321 |
| Molecular Function | Notch binding | 2 | 0.010534084 | 1.97740321 |
| KEGG Pathway | Longevity regulating pathway | 6 | 3.40138E-05 | 4.46834422 |
| KEGG Pathway | Longevity regulating pathway - multiple species | 4 | 0.000901452 | 3.04505729 |
| KEGG Pathway | Acute myeloid leukemia | 4 | 0.001206801 | 2.91836439 |
| KEGG Pathway | Neurotrophin signaling pathway | 5 | 0.001420304 | 2.8476187 |
| KEGG Pathway | Non-small cell lung cancer | 4 | 0.001578247 | 2.80182497 |
| KEGG Pathway | Cellular senescence | 5 | 0.004610642 | 2.33623858 |
| KEGG Pathway | Regulation of lipolysis in adipocytes | 3 | 0.006999123 | 2.15495639 |
| KEGG Pathway | Endometrial cancer | 3 | 0.007714377 | 2.11269913 |
| KEGG Pathway | AMPK signaling pathway | 4 | 0.009828906 | 2.00749481 |
| KEGG Pathway | Transcriptional misregulation in cancer | 5 | 0.011124237 | 1.95372975 |
