# Supplementary material for: Osteoarthritis related epigenetic variations in miRNA expression and DNA methylation
Source: BMC Med Genomics. 2023 Jul 11;16:163. doi: 10.1186/s12920-023-01597-6 (PMC10337191; doi:10.1186/s12920-023-01597-6)
Supplement: Supplementary file 3 — Additional file 3: Supplementary Table S3. Hub genes with the top 10 degrees of both high expression with hypomethylation and low expression genes with hypermethylation. [file 12920_2023_1597_MOESM3_ESM.docx]

Supplementary Table S3. Hub genes with the top 10 degrees of both high expression with hypomethylation and low expression genes with hypermethylation.

| **Gene** | **Gene Description** |  | **Degree** |
| --- | --- | --- | --- |
| ***Hypomethylation and High-Expression hub Genes*** | |  |  |
| COL5A1 | collagen type V alpha 1 chain |  | 2 |
| COL6A1 | collagen type VI alpha 1 chain |  | 2 |
| LAMA4 | laminin subunit alpha 4 |  | 2 |
| ST3GAL6 | ST3 beta-galactoside alpha-2,3-sialyltransferase 6 |  | 2 |
| CUX1 | cut like homeobox 1 |  | 1 |
| FAM198B | family with sequence similarity 198 member B |  | 1 |
| FAM20B | family with sequence similarity 20 member B |  | 1 |
| GALNT1 | polypeptide N-acetylgalactosaminyltransferase 1 |  | 1 |
| GALNT7 | polypeptide N-acetylgalactosaminyltransferase 7 |  | 1 |
| KIF5B | kinesin family member 5B |  | 1 |
| ***Hypermethylation and Low-Expression hub Genes*** | |  |  |
| TP53 | tumor protein p53 |  | 15 |
| FOXO3 | forkhead box O3 |  | 12 |
| EIF4EBP1 | eukaryotic translation initiation factor 4E binding protein 1 |  | 7 |
| PRKAG2 | protein kinase AMP-activated non-catalytic subunit gamma 2  1 |  | 6 |
| RXRA | retinoid X receptor alpha |  | 6 |
| PIK3CD | phosphatidylinositol-4,5-bisphosphate 3-kinase catalytic subunit delta |  | 5 |
| PPARD | peroxisome proliferator activated receptor delta |  | 5 |
| TNS1 | tensin 1 |  | 5 |
| PBX1 | PBX homeobox 1 |  | 4 |
| PDK4 | pyruvate dehydrogenase kinase 4 |  | 7 |
