# Supplementary material for: Osteoarthritis related epigenetic variations in miRNA expression and DNA methylation
Source: BMC Med Genomics. 2023 Jul 11;16:163. doi: 10.1186/s12920-023-01597-6 (PMC10337191; doi:10.1186/s12920-023-01597-6)
Supplement: Supplementary file 4 — Additional file 4: Supplementary Table S4. DEGs associated with both specific miRNA and DNA methylation CpG sites between OA and healthy samples. [file 12920_2023_1597_MOESM4_ESM.docx]

Supplementary Table S4. DEGs associated with both specific miRNA and DNA methylation CpG sites between OA and healthy samples.

| **Gene** | **DMP chromosome location miRNA** | | | |
| --- | --- | --- | --- | --- |
| ***Up-regulated genes affected by both low miRNA and hypomethylation*** | | | | |
| SSH1 | cg25270574 | chr12 | Body | hsa-miR-214-3p |
| GALNT7 | cg24556382 | chr4 | Body | hsa-miR-214-3p |
| TREM1 | cg10981439 | chr6 | 5'UTR | hsa-miR-654-3p |
| VPS13B | cg09461494 | chr8 | Body | hsa-miR-6779-5p |
| VPS13B | cg17117823 | chr8 | Body |  |
| ***Down-regulated genes affected by both high miRNA and hypermethylation*** | | | | |
| ALCAM | cg24273728 | ch3:106567961-106569640 | Body | has-miR-449a |
| ZHX2 | cg00415665 | ch8 | 5'UTR | hsa-miR-1287-5p |
| MIER2 | cg08888178 | chr19:345260-345590 | TSS200 | has-miR-449a |
| IRAK3 | cg18177616 | chr12:66582695-66583345 | TSS1500 | has-miR-449a  7 |
| GDF11 | cg22950598 | chr12:56136783-56137784 | Body | hsa-miR-1287-5p |
| PARD3 | cg14740976 | chr10 | Body | hsa-miR-5581-5p |
| PARD3 | cg26988617 | chr10 | Body | hsa-miR-5581-5p |
| SVIL | cg02153747 | chr1:112524468-112525385 | 5'UTR | has-miR-449a |
| ASPH | cg05142030 | chr1:112531559-112533773 | Body | has-miR-218-5p |
| FOXN3 | cg24714561 | chr14 | 5'UTR | \| has-miR-449a \| \| --- \| \| has-miR-218-5p \| \| hsa-miR-5581-5p \| |
| FOXN3 | cg27293090 | chr14 | Body |  |
| DNMT3A | cg02746110 | chr2 | 5'UTR | has-miR-218-5p |
| DNMT3A | cg02118630 | chr2 | 5'UTR |  |
| DNMT3A | cg23042148 | chr2:25499763-25500429 | Body |  |
| TEX264 | cg19095143 | chr3 | Body | has-miR-449a |
