# Supplementary material for: Osteoarthritis related epigenetic variations in miRNA expression and DNA methylation
Source: BMC Med Genomics. 2023 Jul 11;16:163. doi: 10.1186/s12920-023-01597-6 (PMC10337191; doi:10.1186/s12920-023-01597-6)
Supplement: Supplementary file 5 — Additional file 5: Supplementary Table S5. The gene list of dual-regulated genes. Left: up-regulated genes with hypomethylation and low miRNA. Right: down-regulated genes with hypermethylation and high miRNA. [file 12920_2023_1597_MOESM5_ESM.docx]

Supplementary Table S5. The gene list of dual regulated genes. Left: up regulated genes with hypomethylation and low miRNA.

Right: down regulated genes with hypermethylation and high miRNA.

| Up regulated | Down regulated |
| --- | --- |
| SSH1 | ALCAM |
| GALNT7 | ZHX2 |
| TREM1 | MIER2 |
| VPS13B | IRAK3 |
|  | GDF11 |
|  | PARD3 |
|  | SVIL |
|  | ASPH |
|  | FOXN3 |
|  | DNMT3A |
|  | TEX264 |
